# Can Equids Be a Reservoir of *Leishmania braziliensis* in Endemic Areas?

**DOI:** 10.1371/journal.pone.0093731

**Published:** 2014-04-10

**Authors:** Jessé Henrique Truppel, Flavio Otomura, Ueslei Teodoro, Rubens Massafera, Magda Clara Vieira da Costa-Ribeiro, Carolina Motter Catarino, Luana Dalagrana, Maria Eugênia Moreira Costa Ferreira, Vanete Thomaz-Soccol

**Affiliations:** 1 Center for Zoonoses Control, Department of Health Surveillance, Municipal Health Department, Araucária, Paraná, Brazil; 2 Post-Graduation Program in Microbiology, Parasitology and Pathology, Department of Pathology, Federal University of Paraná (UFPR), Curitiba, Paraná, Brazil; 3 Post-Graduation Program in Health Sciences, Maringá State University (UEM), Maringá, Paraná, Brazil; 4 Surveillance Service of Health, Ministry of Health, Distrito Federal, Brazil; 5 Geography Department, Maringá State University (UEM), Maringá, Paraná, Brazil; Royal Tropical Institute, Netherlands

## Abstract

In this study, we detected *Leishmania* (*Viannia*) *braziliensis* infection in equids living in endemic regions of cutaneous leishmaniasis. To determine the role of these animals in the *Leishmania* cycle, we used two approaches: serological and molecular methods. Antibodies to the parasite were assayed using the Enzyme Linked Immunosorbent Assay (ELISA). Blood samples were collected and tested by polymerase chain reaction (PCR), and the positive products were sequenced. The results showed that 11.0% (25/227) of the equids were seropositive for *Leishmania* sp, and 16.3% (37/227) were PCR positive. Antibodies were detected in 20 horses, 3 donkeys, and 2 mules, and the parasite DNA was detected in 30 horses, 5 donkeys, and 2 mules. Sequencing the amplified DNA revealed 100% similarity with sequences for *Viannia* complex, corroborating the results of PCR for *L. braziliensis*. Our results show that equids are infected with *L. braziliensis*, which could be food sources for phlebotomines in the peridomiciliary environment and consequently play a role in the cutaneous leishmaniasis cycle.

## Introduction

In the New World, cutaneous leishmaniasis in humans is caused by various species, including Leishmania (Viannia) braziliensis, Leishmania (V.) peruviana, Leishmania (V.) guyanensis, Leishmania (V.) panamensis, Leishmania (V.) shawi, Leishmania (V.) lainsoni, Leishmania (V.) naiffi, Leishmania (Leishmania) amazonensis, Leishmania (L.) mexicana, and Leishmania (L.) venezuelensis, while visceral leishmaniasis (VL) is caused by only one species, Leishmania (Leishmania) infantum [1], [2], [3], [4].

The parasite can infect humans in the tropics and subtropics worldwide. The life cycle of *Leishmania* basically involves wild animals and phlebotomines. Human outbreaks have been detected only in extractive activities, deforestations, construction of highways, petroleum exploration, and during scientific exploration [5], [6], [7], [8].

During the last 60 years, in some regions of Brazil, particularly in the southeast and south, there have been extensive changes in the environment and increases in agricultural and pastoral activities. This resulted in the virtual disappearance of cutaneous leishmaniasis (CL) from the end of the 1950s to the 1970s. However, in the 1980s, an increased incidence of CL was observed not only in endemic areas but in new areas, and outbreaks were also reported in regions where leishmaniasis was considered eradicated [9], [10], [11], [12], [13], [14].

Cutaneous leishmaniasis is strongly associated with environmental factors, and its anthropogenic modifications would be considered as modifying actions of risk factors for transmission of the infection. To identify the key and more easily modified factors that prevent/promote the transmission of CL, reservoirs should be considered as essential factors in any investigation to establish the risk factors for transmission of the infection. The reservoir has been well established for numerous *Leishmania* species. However, even though *L. braziliensis* is found throughout the neotropics, its epidemiology is poorly understood. This is because when an infected animal is diagnosed, the strain generally is not isolated and identified, so questions remain regarding the primary reservoir and other potential reservoirs of *L. braziliensis* [5], [14], [15]. Domestic animals, such as dogs and equines, probably play a role in the transmission of *L. braziliensis* since they are closely related with humans and are also present in agricultural and pastoral areas that are generated by deforestation.

Infection of *L. braziliensis* in a horse was first reported in 1927 in an animal from Argentina and in 1959 in a donkey from Brazil [16]. Currently, *Leishmania* infection in horses in the New World has been reported in Brazil, Venezuela, Puerto Rico, and the United States [9], [10], [13], [15], [17], [18].

In the New World, the leishmaniases may be mutating from exoanthropic zoonoses to anthroponoses [6], or euzoonoses, with domestic animals playing a fundamental role in the maintenance of the parasite. To answer this question, our group investigated *L. braziliensis* infection in equids from an endemic rural area of southern Brazil using serological and molecular methods as tools in order to determine if the infection is widely distributed in this animal species in endemic areas.

## Materials and Methods

### Ethics statement

All experiments conducted in the present study comply with current Brazilian laws. All equids owners provided consent to have their animals sampled. None of the animals sampled had clinical signs of leishmaniasis, and all of them had good general health. The equids were submitted only for sample collection, and none of them were subjected to any treatment or invasive procedure. The present study and its animal protocol were approved by CNPq (National Council for Scientific and Technological Development), process number: 301906/2008-4.

### Study area

**Figure 1:**
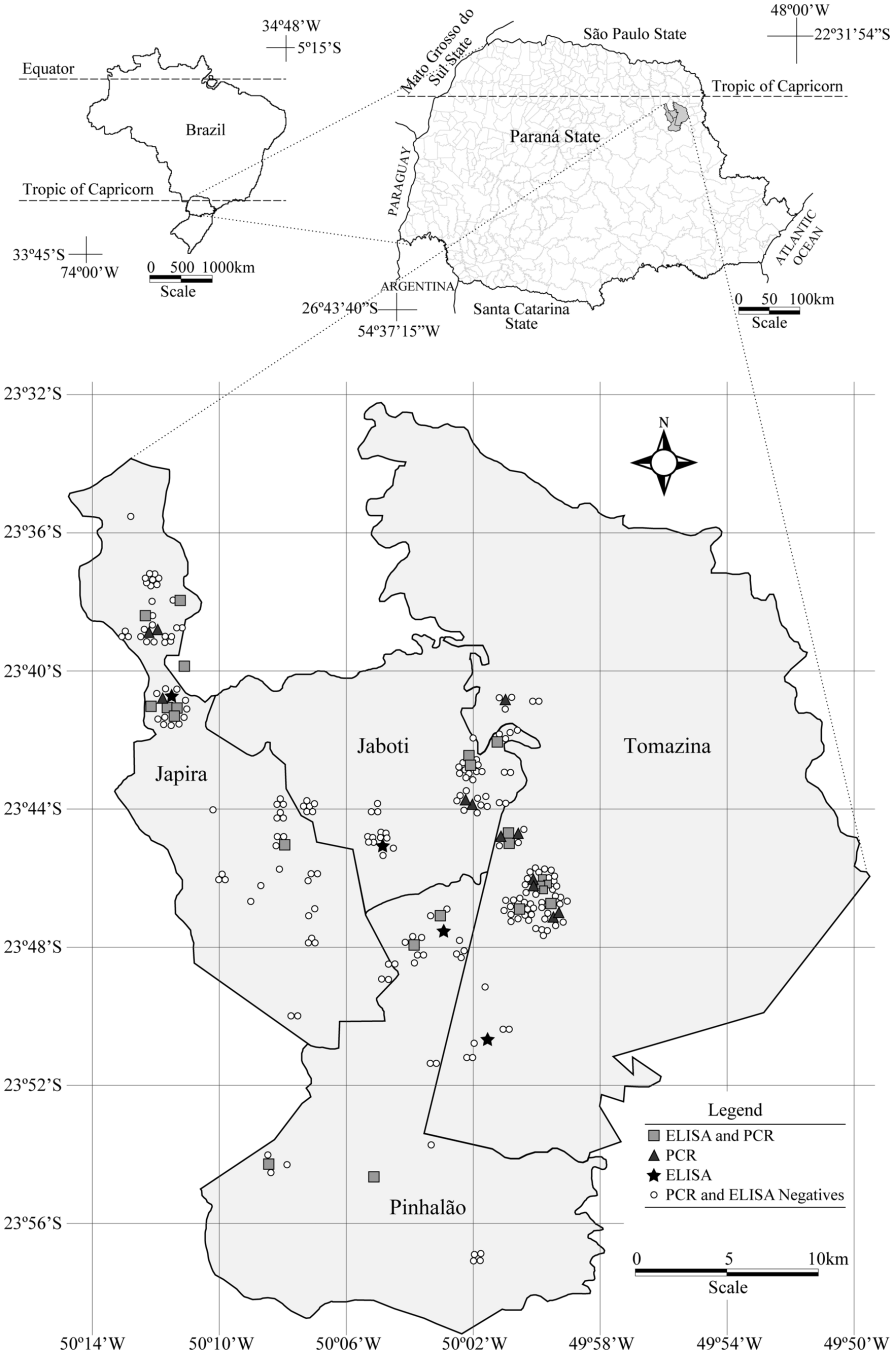
Representative figure of the study area, State of Paraná, southern Brazil, and presentation of the results obtained by ELISA and PCR in their respective localities.

The equids used in the present study were from the counties of Jaboti (23°44′34″S, 50°04′33″ W), Japira (23°48′46″S, 50°08′20″ W), Pinhalão (23°47′34″S, 50°03′21″ W) and Tomazina (23°46′40″ S, 49°57′00″ W). These counties are inserted in the mid-region of northern Paraná State, the Second Paraná Plateau, and the Eastern Portal of the Paraná Basin, with altitudes from 550 to 650 m, between 22°29′23″ and 26°42′59″ south latitude and between 48°02′24″ and 54°37′38″ west longitude (Figure 1).

The study area is defined climatically as **Cfa**, where **C** means average of the coldest month is less than 18°C; **f** means there is no dry season; and **a** indicates a hot summer. The annual rainfall is 2000 mm, distributed throughout the year, with 120 days of rain concentrated mainly in the months of November to January. Since our objective was to analyze the role of equids as reservoirs for *L. braziliensis*, studies were conducted in the four counties mentioned because of the incidence of human cases of cutaneous leishmaniasis occurring there, a reliable indicator for the presence of this parasite.

### Reservoir survey and blood collection

Blood samples were collected from the jugular vein (10 mL) of 227 equids (181 horses, 27 donkeys, and 19 mules) from the counties mentioned above. From these samples, 5 mL were collected in BD Vacutainer tubes to collect the sera for serological assay, and 5 mL were collected in BD Vacutainer spraycoated K_2_EDTA tubes for DNA extraction of whole blood.

### Enzyme-linked immunosorbent assay (ELISA)

The prevalence of anti-*Leishmania* sp. antibodies was evaluated using the enzyme-linked immunosorbent assay antibody (ELISA) test. Sera were submitted to two ELISA steps; in the first (ELISA I), *Leishmania infantum* lysate was used as antigen (1 μg/well), while in ELISA II, the protein rk 39 was used as antigen (25 ng/well). Antigens were purchased from CORIXA CORP, Seattle, WA, USA. The conjugate was anti-horse antibody (IgG-fraction) from sheep, A-6917 (SIGMA™), diluted to 1∶10,000 in PBS buffer (0.01 M, pH 7.2), 1% of Tween 20, and 0.1% of BSA. All samples were analyzed at a 1∶100 dilution in PBS, pH 7.2. The substrate used was TMB Microwell Peroxidase Substrate (Kirkegaard & Perry Laboratories). The cutoff value was obtained from the mean absorbance of 30 negative horse serum samples plus 3 standard deviations. The negative animals came from Curitiba, not an endemic area for L. braziliensis. The ELISA test was performed as proposed by the manufacturer, and the absorbance was under the cutoff value.

### DNA extraction and polymerase chain reaction (PCR)

Blood samples were submitted for DNA extraction using the phenol/chloroform protocol, according to [19], with the following modifications. Samples were centrifuged for 15 min at 3500 *g*, the supernatant was discarded, and the sediment was washed with PBS. Next, lysozyme, 10% SDS, and RNAse were added, and the mixture was homogenized and incubated at 37 °C for 2 h. After this, proteinase K was added, homogenized, and incubated at 55 °C for 2 h. The subsequent extraction was performed by the phenol/chloroform-based protocol [20]. The final extract was air-dried and resuspended in TE buffer (Tris HCl 10 mM, EDTA 1 mM, pH 8.0). This solution was stored at -80 °C.

Subsequently, blood DNA samples were assayed for the presence of *Leishmania* sp. by a single PCR program using the primer pair MP1L and MP3H (5 mM). Amplification reactions were performed in a total volume of 25 μL, with the primary mixture containing 2.5 μL of 10× DNA polymerase buffer (100 mM Tris–HCl, pH8.3, and 500 mM KCl); 1.5 mM MgCl_2_; 0.8 mM of each deoxyribonucleotide triphosphate (dNTPs); 0.1% of Triton-100; 0.5 μL of each primer; 0.2 μL of *Taq* DNA polymerase (InvitrogenTM); and 2 μL of template genomic DNA. Reaction cycles comprised an initial denaturing step at 94 °C for 2 min, followed by 30 cycles of amplification, each cycle consisting of: 94 °C for 1 min, 58 °C for 1 min, and 72 °C for 1 min, with a final extension step at 72 °C for 10 min. Negative and positive controls were included with all PCR reactions, performed in a Hybaid GeneAmp thermocycler with a heated lid. The amplification products were electrophoresed in a 1.6% (w/v) agarose gel, stained with ethidium bromide, illuminated under UV, and photographed with a Life Technologies Gibco BRL UV transilluminator documentation system.

### Sequencing

Five amplified PCR products from each municipality were submitted to sequencing using the Dyenamic ET to MegaBACE (GE Healthcare Life Sciences of Brazil) to confirm the identity of *L. braziliensis*. The sequencing reactions were performed using 100 ng of template amplicons, 3 μL of Dyenamic ET, 1.5 μL of the primer (10 pmol/μL), and 1.5 μL of the appropriated sequencing buffer. The sequencing program comprised 25 cycles of 95 °C for 20 s, 50 °C for 15 s, and 60°C for 1 min, following which the products were precipitated and purified as described by [21]. Consensus sequences were produced using the EMBOSS GUI package software (http://bips.u-strasbg.fr/EMBOSS), the sequenced fragments were aligned with available data deposited in GenBank, and comparisons were performed using BLAST programs available on NCBI to confirm the results (http://blast.ncbi.nlm.nih.gov/Blast.cgi).

### Data analysis

The data were statistically analyzed using the Fisher exact test to determine differences in prevalence between sex groups and equine species (horses x donkeys and mules). A value of P≤0.05 was considered statistically significant. To compare groups (sex and species), we considered the positive results for both PCR and ELISA assays.

## Results

### ELISA

Anti-*Leishmania braziliensis* antibodies were detected in sera from 25 of the 227 equids analyzed (11.0%). Of these, antibodies were identified in 20 horses (11.0%), 3 donkeys (11.1%), and 2 mules (10.5%). Positive sera were detected in 12.2% (6/49) of equines from Tomazina; in 11.5% (9/78) from Japira; 10% of animals were positive from Jaboti (6/60) and Pinhalão (4/40). The seroprevalence in female equines, 13.7% (13/95), was not significantly different from that of male equines, 9.1% (12/132), (*P* = 0.19). None of the animals that were investigated presented cutaneous lesions attributed to *L. braziliensis*.

### PCR and sequencing

Of the 227 equids analyzed, 37 (16.3%) were positive for *Leishmania* sp. DNA. Among these positive animals, parasite DNA was detected in 30 horses (16.6%), 5 donkeys (18.5%), and 2 mules (10.5%). The highest prevalence was determined for the municipality of Tomazina, where 20.4% of the equids were infected (10/49), followed by a 16.7% rate of infection in Jaboti (10/60), 15.0% in Pinhalão (6/40), and 14.1% in Japira (11/78). Fifteen PCR fragments were sent out for sequencing, and all shared 100% similarity with sequences for *Viannia* complex, corroborating the PCR results for *L. braziliensis*.

### ELISA and PCR

Analyzing ELISA and PCR, past or current infection with *L*. *braziliensis* was detected in 10.1% (23/227) of the animals studied considering both ELISA and PCR results and in 17.2% (39/227) of the equids positive for either ELISA or PCR (Table 1). Regarding sex, considering the results for both tests, we verified that 9.1% (12/132) of male equids and 11.6% (11/95) of female equids were positive for *L*. *braziliensis*. Analyzing the PCR and ELISA data, we determined that there were no significant differences in the infection rates between species (*P* = 0.52) and sexes (*P* = 0.37).

**Table 1:** The prevalence of *L. (V.) braziliensis*-positive equids (horses, donkeys, and mules) determined by ELISA and PCR.

	Number of positive equids/species analyzed (rate of infection)			Total of positive equids/animals analyzed (rate of infection)
Method	Horse	Donkey	Mule	TOTAL
**ELISA**	20/181	3/27	2/19	25/227
	(11.0%)	(11.1%)	(10.5%)	(11.0%)
**PCR**	30/181	5/27	2/19	37/227
	(16.6%)	(18.5%)	(10.5%)	(16.3%)
	Positive equids in both tests, ELISA and PCR/equids analyzed (rate of infection)			23/227 (10.1%)
	Total of positive equids in either ELISA or PCR/equids analyzed (rate of infection)			39/227 (17.2%)

## Discussion

This study proved that equids (horses, donkeys, and mules) from southern Brazil are infected with *L. braziliensis*, since antibodies were detected in sera and parasite DNA were found in the blood samples. The sequencing confirmed that the parasite was *Viannia* complex, thus corroborating the presence of *L. braziliensis*. *Leishmania* DNA in the blood was observed in 16.3% of the animals, and 11.0% presented positivity by ELISA. A positive ELISA with negative PCR is obtained in cases when the equid has only been exposed to the parasite in the past or when the parasite is in the chronic phase of infection and not present in the blood. In addition, a positive PCR with negative serology is obtained in cases of acute infection, since we only analyzed IgG antibodies, and when the animal has some immune deficiency disease.

The presence of anti *L. braziliensis* antibodies is evidence that the immune system had prior contact with the protozoan, while the presence of specific DNA confirms blood circulation of the parasite. We detected *L. braziliensis* DNA in 23 of the 25 equids with positive sera, while 14 PCR-positive animals had no antibodies against the parasite. These data could be explained by the possibility that we detected *L. braziliensis* in the acute phase of infection. These results are evidence of the presence of the parasite and of the peridomiciliary transmission of this parasite in the study area (Figure 1).

Since the first report in Argentina, studies have developed the possibility of horses, donkeys, or mules being accidental hosts or reservoirs or even having a role in the cycle of *Leishmania* sp and CL in the peridomiciliary environment (Table 2). The main epidemiological studies addressing the occurrence of CL in horses have been conducted in South America [3], [10], [15], [18], [22], [23]. Because of the presence of horses infected with *Leishmania* in endemic areas for leishmaniasis in humans, these authors discussed the chance that horses could take a part in the maintenance cycle of the parasite in this transitional environment between wild and domestic habitats. In studies conducted by [10], [18], [22], [23], the parasite was isolated and identified as *Leishmania braziliensis*. Most studies of leishmaniasis in horses have been conducted in South America, particularly in Brazil [3], [10], [13], [15], [17], [18], [25], [28]. There are case reports of CL in two horses in Central America [24] without *Leishmania* species identification and an infected horse in North America [27], in which the identified species was *L. siamensis*. Case reports have also been published in Europe; in Germany [26] and in Spain [27] *L. infantum* was identified as the causative agent for cutaneous lesions in horses. In recent epidemiological studies conducted in Brazil, [13], [25] investigated the presence of *Leishmania* in 132 horses. In both studies, none of these animals presented cutaneous lesions. In a serological survey performed in an area endemic for CL in humans, [25] found 22.1% of horses carried antibodies against *Leishmania* sp. In the study conducted by [13], of the 55 horses analyzed, 76.3% were seropositive for *Leishmania* sp, and 7.1% were PCR positive in blood samples. The parasite was not isolated or sequenced in either study [13], [25]. In our study, 227 equids were analyzed. It is therefore the largest epidemiological survey regarding leishmaniasis and horses in a transmission environment for CL. All horses evaluated in this study were present in areas endemic for CL. During the sample collection, no humans presented cutaneous lesions. Similarly, none of the horses, donkeys, or mules had cutaneous lesions. Our results showed that 25 of the 227 equids had IgG antibodies against *Leishmania* sp. And, of these 25 seropositive animals, 23 the parasite was present in the blood circulation, as their blood samples were PCR positive. This finding may suggest the possibility of these infected equids not only to serve as a food source for sand flies but also to maintain the life cycle of *L. braziliensis* in peridomiciliary areas. This result is unprecedented in identifying blood circulation of *L. braziliensis* (PCR and sequencing) in seropositive horses (IgG ELISA). In this way, horses could act as infection sources for sand flies and contribute to this epidemiological context in that CL is characterized by synanthropic zoonoses. In order to prove that these animals can act as domestic reservoirs of *L. braziliensis* in the peridomiciliary environment is necessary to conduct infectivity tests on sand flies and perform the isolation of the parasite from samples of peripheral blood.

**Table 2:** References to studies on cutaneous leishmaniasis and horses, showing the numbers of animals evaluated and the results obtained.

Country	Authors Year	Equids analyzed	Cutaneous lesions	*Leishmania* sp. direct detection^1^	Serology	PCR (target primers)	Isolation^2^	Sequencing
Venezuela	[22] 1984	29	Present	21.4%	-	-	*L. braziliensis*	-
Brazil	[3] 1986	26	Present	30.8%	-	-	-	-
Brazil	[15] 1987	14	Present	7.1%	-	-	-	-
Brazil	[10] 1988	26	Present	30.8%	-	-	*L. braziliensis*	-
Brazil	[18] 1989	32	Present	28.1%	-	-	*L. braziliensis*	-
Brazil	[17] 1990	1	Present	100%	-	-	*L. braziliensis*	-
Venezuela	[23] 1992	5	Present	100%	-	-	*L. braziliensis*	
Puerto Rico	[24] 1996	2	Present	100%	-	-	-	-
Brazil	[25] 1999	77	Absent	-	22.1%	-	-	-
Germany	[26] 2002	1	Present	100%	0%	100% (*L. infantum*)^3^	-	-
Spain	[27] 2003	3	Present	100%	33.3%	-	*L. infantum*	-
Brazil	[13] 2008	55	Absent	-	76.3%	7.1% (*L.braziliensis*)	-	-
USA	[28] 2012	1	Present	100%	-	100% (*Leishmania sp.*)	-	*L. siamensis*
Brazil	[29] 2013	3	Present	66.7%	66.7%	100% (*L. braziliensis L. infantum*)	-	-
Brazil	In this study	227	Absent	no lesions	11.0%	16.3% (*L. braziliensis*)	-	*L. braziliensis*

1 ***Leishmania*** **sp. direct detection**: cytology or histology and staining, microscopic diagnostic.

2 **Isolation of the parasite**: species identification by biochemical characterization (isoenzyme pattern), monoclonal antibodies, zymodeme and serodeme analysis.

3 *L. infantum* identified by restriction fragment length polymorphism (RFLP).

In rural areas of Brazil, equids are commonly used as means of transport, as pack animals in deforestation areas, and in agropastoral activities, therefore they are constantly present in endemic areas and can become a good blood source for phlebotomines [3]. This change in the habits of sand flies results from the human-induced environmental change in order to increase agricultural and pastoral areas. As a result of deforestation and human migration to these new agropastoral regions, where the population has built precarious housing on the edges of preserved woods and secondary woods, phlebotomines have adapted to the peridomiciliary environments. This altered behavior in relation to food sources might be a future adaptation of sand flies, since studies show that *Lutzomyia* (*N*.) *intermedia* feeds on people, rodents, birds, horses, and dogs [30]. Since this feeding habit is directly related to human activities, phlebotomines may have adapted to new ecotopes in the peridomiciliary environment, resulting in new epidemiological patterns for CL, thus creating a favorable eco-epidemiological scenario [6], [20].

Studies performed in the State of Paraná have revealed high numbers of phlebotomines collected in houses and shelters near deforestation areas and in secondary woods in CL endemic regions, proving the tendency of peridomiciliary behavior of sand flies in these locations [31], [32]. Our concern regarding this epidemiological context is to identify the reservoir(s) that could maintain the life cycle of *L. braziliensis* in the peridomiciliary environment, since in any epidemiological research, knowledge regarding the reservoirs, phlebotomines, and leishmanial parasites in any disease foci is essential for correct diagnosis and treatment and precise control strategies [14], [33]. Regarding this scenario, where sand flies appear to be adapting to new ecotopes and different food sources, both close to anthropic environments, consequent pressure is being exerted on the *Leishmania* species, particularly on *L. braziliensis*. This parasite species presents a high degree of genetic variability and thus could infect a larger number of vectors and mammals, permitting the appearance of new transmission areas with different species of vectors and reservoirs [34], [35], [36], [37], [38]. Studies have shown genetic polymorphism in natural populations of different species of *Leishmania* sp. [34], [35], which could explain the plasticity of these parasites and their ability to adapt to new ecological conditions [6].

In the New World, the identification of the primary reservoirs (wild animals x domestic animals) in the maintenance of the *L. braziliensis* cycle is still under investigation. It is known that CL is a zoonoses that was originally restricted to the sylvatic cycle and that human-induced environmental changes in rural and urban areas brought the parasite into the domestic cycle, increasing the incidence of human and domestic animals (dogs and horses) infected with *L. braziliensis* [11]. A high prevalence of domestic animals infected with *L. braziliensis* is usually related to epidemic outbreaks, when the same infection pressure suffered by humans is observed among dogs or horses, which show similar or lower seroprevalence. A study conducted in Paraná state, [14], discussed the role of dogs and equids in the transmission of *L. braziliensis* based on serology, anatomopathological tests and *in vitro* culture. It was not possible to isolate the parasite from intact skin, blood, lymph nodes, or internal organs of any of the equids, later diagnosed as *Habronema* sp.

The Koch postulates are partially fulfilled and have shown that equids (horses, donkeys, and mules) are infected with *L*. *braziliensis*. Therefore, these animals can be sources of food for the sand flies outside the home, allowing maintenance of the peridomestic cycle of the parasite and thus contributing to the epidemiology of human cutaneous leishmaniasis.
